# Forum theater staging of difficult encounters with patients to increase empathy in students: evaluation of efficacy at The University of Angers Medical School

**DOI:** 10.1186/s12909-020-1965-4

**Published:** 2020-02-24

**Authors:** Marion Sevrain-Goideau, Benedicte Gohier, William Bellanger, Cedric Annweiler, Mario Campone, Regis Coutant

**Affiliations:** 1 Department of Pediatrics, University Hospital, 4 rue Larrey, 49000 Angers, France; 2 Department of Psychiatry, University Hospital, Angers, France; 3 Medical School, University of Angers, Angers, France

**Keywords:** Communication skills, Ethics/attitudes, Medicine, Simulation

## Abstract

**Background:**

Physician empathy has been associated with improved clinical outcomes and lower physician burnout. We evaluated whether forum theater (FT), a form of applied drama that allows participants to enter the performance and represent the actions associated with emotions, would foster empathy in medical students, and which underlying variables would be associated to empathy scores.

**Methods:**

Three classes totaling 488 fourth-year medical students participated in the study. Forum theater was used to explore difficult encounters with patients and family members: announcement of cancer, fall at home of an elderly person requiring hospitalization, appointment with a patient suffering from depression, announcement of diabetes in an adolescent. The first scene was played by actors in front of a group of students, then audience members were asked to enter the performance and, by taking over the role of the “physician-actor,” to explore alternative interactions. All the students followed two sessions as actors and observers in random order and were randomly assigned to FT sessions after 36 or 56 weeks of clinical rotations. They completed the Jefferson Scale of Physician Empathy (JFSE) anonymously.

**Results:**

Students were 22.1 ± 1.5 years old (43% males). Empathy scores increased after each session: 102.0 ± 9.8 before the sessions, 106.3 ± 9.8 after session 1 and 107.8 ± 11.5 after session 2 (*p* <  0.05). In regression models, gender (F vs. M, + 3.0 ± 1.0, *p* <  0.001) and position in the session (actor vs. observer, + 2.1 ± 1.0, *p* < 0.05) were significant determinants of JFSE scores, whereas age, session theme, and duration of clinical rotation were not.

**Conclusion:**

Being an actor in forum theater was a valuable tool for enhancing empathy scores in medical students.

## Background

There is a long tradition of research regarding physician’s empathy, including definitions and measurements. Several thousands of papers dealing with empathy in physicians and medical students have been published [1]. Empathy is considered as a highly desirable competence, even rated as one of the most important by medical students, physicians, and patients [1]. In their Learning Objectives for Medical School Education, the Association of American Medical Colleges states that “physicians must be compassionate and empathetic in caring for patients” [2]. This statement illustrates a commitment to producing not only the most knowledgeable and skillful physicians possible, but also the most caring. Although the precise definition of empathy is a matter of debate, most constructions of empathy have in common the caregiver’s cognitive and vicarious understanding of the patient as a person, including the emotional states, and the expression of this understanding [3–5]. Physician empathy has been associated with higher patient satisfaction, adherence to medical recommendations, and improved clinical outcomes, and with lower physician burnout, higher well-being, higher ratings of clinical competence, and less medical-legal risk [1]. Physician empathy may even reduce healthcare costs [1].

For all these reasons, there are increasing calls to assess the level of physician empathy, especially since some studies, though not all, have shown a decline in empathy during medical education [6]. In a systematic review of tests of empathy in medicine, Hemmerdinger et al. [4] retained eight instruments with evidence of reliability, internal consistency, and validity. Among them, the most frequently used was the Jefferson Scale of Physician Empathy (JFSE), a self-rating test that has correlated with assessed competence in students, later ratings of empathy by directors during residency, and patient-report measures of physician empathy [3, 4, 7–10].

Despite the undeniable importance of these skills to successful medical practice, no widespread or well-studied curricula exist to teach clinical empathy. In two recent systematic reviews of interventions to cultivate physician empathy, Kelm et al. [1]. selected 64 studies that quantitatively assessed changes in empathy, and Patel et al. [11] selected 52 studies that were controlled. More than half have been directed toward residents or physicians, that is professionals advanced in their medical training and already engaged in a specialty, which may be considered as relatively late in the curriculum [1, 11]. The sample size was 11 to 439 subjects (median 78), the interventions lasted 40 min to 96 h (median 12 h), and 42 studies (66%) reported a significant increase in empathy [1]. Interventions included communication skills training interventions (using such didactic materials as lectures, handouts, and audio- and videotapes), drawing on the humanities (using reflective writing and theater), interviews of standardized patients, role playing interventions in which participants act as patients or family members, and mindfulness-based stress reduction interventions [1, 11, 12].

Among the tools designed to build an understanding of how a person experiences others, theater and applied drama have been used to bridge the gap between theoretical knowledge and practice. Theatrical performances, well-known dramas as “Wit” [13], or other staged performances to present the patient experience of illness [14, 15], have been used for audience members (medical students) to discuss their reactions and feelings toward the plot, allowing an ethical reflexion to occur [13, 14], and/or allowing to build communication skills [15]. Other forms of theater required the active engagement of the participants. It allows them to act out experiences and situations in order to better understand illness from the patient point of view, emotions of others, as well as the complex interactions that occur during the act of delivering and receiving bad news. Improvisational theater, or role-play with standardized or simulated patients, have been used in medical students or residents, to teach communication skills [16, 17]. Forum theater (FT), introduced in the 1970s by Augusto Boal, is a form of applied drama where an issue or dilemma is used for exploration by a small group of participants (as actors) in front of a larger group of peers, as a way of exploring solutions to real-life dilemmas in a safe environment [18, 19]. The audience members in FT (whom Boal calls “spect-actors”) are asked to enter the performance and, by taking over the role of one of the “actors,” to explore alternative interactions. Forum theater is an experiential theatrical technique that directly involves students as spect-actors, enabling them to explore and practice multiple ways of communicating without resorting to any kind of prescriptive answer for a given situation. These interactions may lead to a more positive outcome than the one presented in the original scenario. The purpose is to stimulate discussion and interactive reflection on the dramatized scenarios [19]. In healthcare education, FT has been used with students from nursing, midwifery and medicine to investigate issues that students may find challenging, such as empowerment, valuing diversity and exploring values and beliefs [19, 20]. To our knowledge, FT has not been evaluated as a tool to increase communication skills or empathy scores among medical students up to now.

In the present study, we explored the use of FT to foster communication skills and empathy in the fourth-year medical students of Angers Medical School. Our first objective was to evaluate whether FT increased empathy, as measured by the JSPE before and after FT in a class of 180 fourth-year medical students. Our secondary objective was to explore the determinants of empathy measured after FT in a larger group of 488 fourth-year medical students from three consecutive classes.

## Methods

### Participants

Participants were fourth-year medical students from Angers Medical School from classes 2014–2015 (*n* = 193), 2015–2016 (*n* = 191), and 2016–2017 (*n* = 195), totaling 579 students. Fourth-year medical students in France are 22 years of age on average and have a mix of medicine courses and of real-life encounters with patients under supervision during this year. Medical schools offer a 6 years curriculum (corresponding to both undergraduate and medical studies in other countries) before entering residency. The first two years of medical school consists of basic science courses, and from the third to the sixth year of clinical rotations in teaching hospital (every morning from 9 am to 1 pm) and clinical medicine courses in the medical school (every afternoon from 2 pm to 6 pm). Then, sixth year medical students undergo a national written exam for entering residency, which last 4 to 6 years, depending on the specialty. We therefore estimated that fourth-year medical students had the necessary background and the appropriate environment for their ethical reflection to be contextualized.

### Study design

In the first class (*n* = 193), all the students were invited to enroll in the study and complete the JSPE before FT sessions. They were then randomized to follow FT sessions after a total of 36 weeks of clinical rotations under the supervision of residents and associate professors or after a total of 56 weeks of clinical rotations (that is 20 weeks later). Each student participated in two sessions of FT (see below) within 2 weeks and completed the JSPE at the end of each session. This design was compatible with the fourth-year curriculum. To further study the determinants of empathy scores, fourth-year students from the next two classes were invited to complete the JSPE at the end of each session of FT, according to the same randomization process as described above.

### Forum theater

Each student had 12 h of FT training. First, a 2-h lecture including videotapes was given to the students to explain the principles of medical communication according to the Health Professions Core Communication Curriculum [21], to provide information on how to break bad news [22], and to present the briefing for the FT sessions [23, 24]. Two hours of personal homework were expected from each student, but not assessed before FT sessions.

Each student then followed two 4-h sessions of FT (8 h in total). One session comprised three scenarios of an evolving situation of a patient with cancer, and one session comprised three different and independent scenarios. The students attended the sessions in random order. The precise content of the scenarios is detailed in Additional file 1. The first scenario was played out in front of a group of 18 students by actors in amateur theater, including healthcare professionals (nurses, physicians), with one playing the patient, one a family member or caregiver, and one the attending physician whose obvious mistakes in communication bordered on caricature. Then, a medical student entered to replace the actor-physician and proposed more appropriate communication. Next, each of the 2 following scenes consisted of a slight evolution of the initial scene (see Additional file 1). The patient’s attitudes and reactions, as well as those of the family member or caregiver, were different in each of the scenes. As a new medical student entered to play the physician in each scene, this led the student to adapt his/her communication skills to the situation. Therefore, for each scenario, three medical students were actors, and for each 4-h session, three scenarios were acted out (therefore 9 students were actors). Moreover, each group of 18 students followed the two different sessions, allowing each student to be an actor at least once and an observer for the other scenes. Each scenario was played out for approximately 30 min, and this was followed by a debriefing for the next 25 min with the student actors, student observers, actors, and two or three teachers competent in simulation and debriefing. The original scenarios had been developed by a group of eight teachers and eight fourth- to sixth-year medical students to stage difficult encounters with patients.

### Data collection

The JFSE was completed anonymously by each participating medical student as explained above. The JSPE is a 20-item questionnaire consisting of a 7-point Likert scale to evaluate physician self-reported clinical empathy. It has been translated into 53 languages and validated in French [25, 26]. The higher the score, the higher the self-reported clinical empathy, with a possible range of 20 to 140. The tentative cutoff score to identify low and high scorers is believed to be ≤95 and > 125 for men and ≤ 100 and > 129 for women [27]. Hojat et al. [9] also described a three-factor solution: “perspective taking,” with ten items loading (score from 10 to 70), “compassionate care,” with eight items loading (score from 8 to 56), and “standing in the patient’s shoes,” with two items loading (score from 2 to 14).

The type of session (cancer vs. other), the order of the sessions (cancer-other, or other-cancer), the classes (2014, 2015, or 2016), the duration of clinical rotations at the time of FT (32 vs. 52 weeks), the role of the student (actor, or observer), age and gender were recorded.

The objectives were: 1) to evaluate whether FT sessions were associated with an increase in the JFSE score in fourth-year medical students; 2) to identify determinants of the JFSE score measured after the FT sessions.

### Statistics

Continuous variables were expressed as means and standard deviations and discrete variables as percentages. In addition to descriptive statistics, we used the χ^2^ test, the two-tailed Student t test and analysis of variance (ANOVA) followed by least significant difference (LSD) post hoc tests to examine associations between the JSPE scores and the criterion measures. Multiple linear regression analyses were performed, with JSPE scores as the dependent variables and age, gender, session of FT (first vs. second), role (actor vs. observer), scenario (cancer vs. other), and class number as independent variables. Statistical significance was defined as *p* < 0.05. Statistical analysis was performed using IBM-SPSS 20 (IBM, Armonk, NY, USA).

## Results

**Fig. 1:**
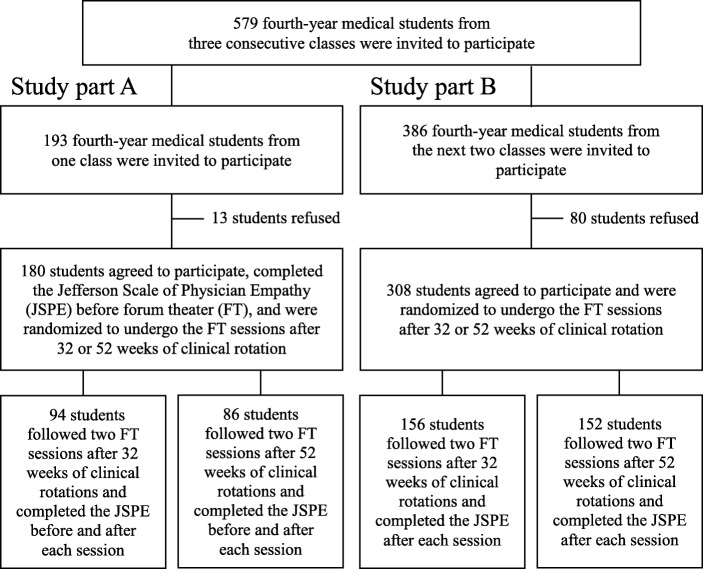
Flow chart of the study

The flow chart of the study is presented in Fig. 1. The mean age of the students was 22.1 ± 1.5 years (20–38 years), and the percentage of male students was 43%.

### Impact of FT sessions on empathy scores

To study the impact of FT on the empathy scores, students from one class (Fig. 1, study part A) completed the JSPE before and after each FT session. The evolution of the scores is described in Table 1. The global JSPE scores were significantly different before and after the first FT session (102.0 ± 9.8 vs. 106.3 ± 9.8, *p* < 0.05) and between the first and second FT sessions (106.3 ± 9.8 vs. 107.8 ± 11.5, *p* < 0.05). All three subscales (“perspective taking,” “compassionate care,” and “standing in the patient’s shoes”) significantly increased after the FT sessions (Table 1). Before the sessions, 32% of the students having an empathy score below the suggested low cutoff (95 in males, 100 in females). This decreased to 16% after the FT sessions (*p* < 0.05).

**Table 1:** Empathy scores on the JSPE before and after the forum theatre sessions

JSPE scores	Forum theater sessions			*P*
	Before	1st session	2nd session	ANOVA
Global	102.0 ± 9.8	106.3 ± 9.8^1^	107.8 ± 11.5^1,2^	< 0.001
Perspective taking	47.8 ± 6.4	48.4 ± 6.9^1^	49.7 ± 7.8^1,2^	< 0.05
Compassionate care	45.1 ± 5.2	47.9 ± 4.4^1^	47.7 ± 5.1^1^	< 0.001
Standing in the patient’s shoes	9.3 ± 2.4	10.1 ± 2.5^1^	10.5 ± 2.4^1^	< 0.001

Differences between the 3 groups by ANOVA

^1^ *p* < 0.05 with the score measured before FT sessions by least square difference post hoc tests

^2^ *p* < 0.05 with the score measured after the 1st FT session by least square difference post hoc tests

### Determinants of the empathy scores after the FT sessions

To further refine the identification of the determinants of empathy scores after the FT sessions, multiple regression analyses were performed using data from the next two classes (Fig. 1, study part B) in addition to the data from the first class in study Part A: in total, 488 out of 579 fourth-year students agreed to participate. The global scores and subscale scores did not differ by ANOVA between the three classes and were therefore grouped in the analyses.

The empathy scores after the first session enabled us to study the impact of the theme of the FT session (cancer vs. other), the position of the students (actor vs. observer), the clinical rotation duration (36 vs. 56 weeks), age and gender (Table 2): global empathy scores were significantly dependent on position (actor vs. observer, + 2.10 ± 0.96, *p* < 0.05) and gender (F vs. M, + 3.04 ± 0.97, *p* < 0.05), but not dependent on theme, class, or duration of the clinical rotations. Of note, one of the three factors of the JSPE, namely “standing in the patient’s shoes,” was dependent on the duration of clinical rotation (36 vs. 56 weeks) (Table 2).

**Table 2:** Multiple regression analyses with the JSPE scores after the first FT session as the dependent variable (*n* = 488)

Dependent variable Empathy scores after the 1st FT session	Variables	Regression coefficient β ± SD	*p*
Global score Multiple R 0.17 *p* < 0.05	Age	−0.18 ± 0.31	NS
	Gender (F vs M)	3.01 ± 0.98	< 0.001
	Position (actor vs. observer)	2.10 ± 0.96	< 0.05
	Theme (cancer vs. other)	−0.29 ± 0.99	NS
	Classes	−0.15 ± 0.58	NS
	Clinical rotation (56 vs. 36 weeks)	0.40 ± 0.98	NS
Perspective taking Multiple R 0.12 *p* = 0.31	Age	0.03 ± 0.21	NS
	Gender (F vs M)	0.83 ± 0.65	NS
	Position (actor vs. observer)	1.47 ± 0.65	< 0.05
	Theme (cancer vs. other)	−0.13 ± 0.66	NS
	Classes	−0.27 ± 0.39	NS
	Clinical rotation (56 vs. 36 weeks)	−0.04 ± 0.66	NS
Compassionate care Multiple R 0.21 *p* < 0.001	Age	−0.18 ± 0.15	NS
	Gender (F vs. M)	1.92 ± 0.45	< 0.001
	Position (actor vs. observer)	−0.36 ± 0.44	NS
	Theme (cancer vs. other)	−0.52 ± 0.46	NS
	Classes	−0.09 ± 0.27	NS
	Clinical rotation (56 vs. 36 weeks)	−0.13 ± 0.45	NS
Standing in the patient’s shoes Multiple R 0.15 *p* = 0.03	Age	−0.04 ± 0.08	NS
	Gender (F vs. M)	0.26 ± 0.23	NS
	Position (actor vs. observer)	−0.27 ± 0.23	NS
	Theme (cancer vs. other)	0.37 ± 0.24	NS
	Classes	0.21 ± 0.14	NS
	Clinical rotation (56 vs. 36 weeks)	0.56 ± 0.23	< 0.05

After the second FT session, all the students had held positions of both actor and observer and had followed the two themes of FT, which were consequently no longer significant determinants of the empathy scores. However, analyses on the empathy scores after the second FT session allowed to study the impact of the order of the two themes (cancer-other vs. other-cancer), together with age and gender. Only gender was a significant predictor of global score of empathy, whereas the order of the themes was not.

## Discussion

We have shown in this study that the global empathy score of medical students was increased by an average of 6 points (from 102 to 108) with the participation to two forum theater sessions, and that the percentage of low-scorers was decreased by half after the two sessions. Consistently, the three meaningful factors of the JPSE (“perspective taking,” “compassionate care,” and “standing in the patient’s shoes”) increased with FT. Significant determinants of empathy scores were gender (female students had higher scores than male students) and the participation as an actor in the FT sessions (as compared to mere observer), whereas clinical rotation duration (except on the factor of “standing in the patient’s shoes”) and the theme of the FT sessions (cancer vs. other) had no impact on the scores.

### Forum theater and empathy

Functional MRI studies have suggested that empathic resonance occurs via communication between action representation networks and limbic areas provided by the insula: to empathize, we need to invoke the representation of the actions associated with the emotions we are witnessing [28]. Empathizing individuals show more neural activity in mirror neuron areas of the brain [29]. As FT directly involves students as spec-actors, this may explain the ability for this kind of applied drama to link with the emotions. On the basis of our findings in the present study, we believe that FT is a powerful tool for developing empathy. Moreover, we observed that empathy scores increased more in actors than in mere observers, which agrees with the functional MRI studies of empathetic resonance and mirroring theories. However, FT implementation was time and human resources consuming and should be carefully weighed against other methods to increase empathy in medical students.

### Empathy scores

We found a mean 3-point difference in the global empathy score in favor of female compared to male students. The gender effect on empathy score has been well-described in numerous studies, and the magnitude of the difference found here was similar to that found in other studies in medical students [3, 9, 10, 27]. Several explanations can be offered for gender differences in empathy. For example, it has been suggested that women are more receptive than men to emotional signals, a quality that can contribute to better understanding and, hence, to a better empathetic relationship. Also, on the basis of the evolutionary theory of parental investment, women are believed to develop more caregiving attitudes toward their offspring than men [9].

The mean empathy score of French fourth-year medical students having a mean age of 22.1 ± 1.5 years was 102.0 ± 9.8 before the FT sessions. This increased to 107.8 ± 11.5 after the sessions, corresponding to 105.0 ± 12.4 and 109.7 ± 10.6 in males and females, respectively. Similar scores have been described in medical students (at approximately the same stage of medical education) from India, the Caribbean, Korea, and Japan, whereas higher scores have been reported in students from the USA, the UK, Australia, New Zealand, and Portugal [30]. For instance, American medical students usually obtain a mean total score of 114 ± 10, higher in women (116 ± 10) than in men (112 ± 10) [20]. The differences between countries may be due to confounding factors, such as course content and structure. Differences between Asian and North American or European medical schools have been ascribed to communication patterns that place less emphasis on nonverbal communication, cultural differences (most Japanese patients prefer their physicians to be calm and un-emotional) [31], and the strongly science-oriented selection system among Asian medical schools [30]. Response bias could also contribute to these differences. On the one hand, social desirability bias describes the risk for a socially desirable “expected response” that students are required to conform to in order to obtain reward (especially in students from medical schools including personality assessment in the evaluation process) [32]. On the other hand, cultural variables around emotional expression, such as the perceived value of modesty, could also explain the differences in empathy scores between countries and cultures. It would therefore be useful to explore the efficacy of FT to increase empathy in different cultures. Notably, the selection process in France is also very strongly science-oriented, based almost exclusively on written exams, with no interview or evaluation of communication skills. However, the JSPE questionnaires were blinded, the questionnaires were completed on a voluntary basis, and participation in the FT sessions was mandatory but not associated with summative evaluation. This may have reduced social desirability bias.

There was no effect of clinical rotations on empathy scores, except for the factor “standing in the patient’s shoes,” which significantly increased with the duration of clinical rotations. The impact of clinical experience on empathy scores has been widely studied, with some studies showing a decline with clinical experience, whereas other studies have shown stability [6, 30, 33]. Here, we compared the effect of short periods of clinical rotation (36 vs. 56 weeks), and we found a positive impact of clinical experience on one of the three factors of the JSPE, namely “standing in the patient’s shoes,” which is a metaphorical component of empathy that provides insight into another person’s thoughts, feelings, and behavior. The theme of the session (one session was dedicated to cancer, with survival and end-of-life issues, whereas the other session was dedicated to less emotional situations) had no impact on empathy scores. This suggests that through FT the medical students were able to separate emotions, affects, and sympathy with the patient from empathy and the understanding of the patient’s state [34].

### Limitations

This study has several limitations. First, the randomization process distributed the students as following FT sessions after either 36 or 56 weeks of clinical rotations but did not distribute students as following or not following the sessions, as all students underwent FT in their fourth year. This was mandatory because this experimental design is permitted in French medical schools provided that in the end all students have similar course content. This design let us study the impact of differences in both clinical rotation durations and FT sessions (and other factors like age and gender) on empathy scores by multiple regression analyses, rather than by direct comparisons. Second, study participation was voluntary, and 486 of 579 students (84%) finally participated. Although we do not know whether the non-participating students had specific characteristics, the absolute number of participants and the 84% participation rate were in the upper range for intervention studies on empathy in medical students, and we believe that the findings of this study are therefore valuable [1]. Finally, empathy was measured immediately after the FT sessions, which may have influenced the scores. It would be useful in future research to explore the long-term impact of the intervention as well as the impact of FT in the empathical behavior observed in real life, that is in clinical practice.

## Conclusions

In conclusion, we showed here that forum theater is a powerful tool for enhancing empathy scores in medical students. The participation as an actor in the sessions (as compared to mere observer), the number of FT sessions, and gender were associated with an increase in empathy scores, whereas clinical rotation duration (except on the factor of “standing in the patient’s shoes”) and the theme of the FT sessions (cancer vs. other) had no impact on the scores. Although the self-rating JFSE scores have been correlated with the assessed competence and ratings of empathy by directors and patients [3, 4, 7–10], the evolution of empathy during medical school, the persistence of the effect of FT across time, and the benefit of FT to foster empathy in later clinical care are still to be demonstrated. Furthermore, one future direction for research might focus on functional MRI studies as they may establish neural correlates with changes in empathy scores following forum theater sessions in medical students.

## Supplementary information

**Additional file 1.** Detailed content of the scenarios.

## Data Availability

The datasets used and/or analyzed during the current study are available from the corresponding author on request.

## Abbreviations

FT: Forum theater
JSPE: Jefferson scale of physician empathy
