# Supplementary material for: Forum theater staging of difficult encounters with patients to increase empathy in students: evaluation of efficacy at The University of Angers Medical School
Source: BMC Med Educ. 2020 Feb 24;20:58. doi: 10.1186/s12909-020-1965-4 (PMC7041274; doi:10.1186/s12909-020-1965-4)
Supplement: Supplementary file 1 — Additional file 1. Detailed content of the scenarios. [file 12909_2020_1965_MOESM1_ESM.docx]

**Additional file 1**

**Detailed description of the scenarios**

Three scenarios were built on the announcement of cancer and treatment for one FT session. In the first scenario, the first scene consisted of an embarrassed practitioner announcing the discovery of a suspicious lesion during a colonoscopy performed the day before in a patient whose mother had died of colon cancer: the anatomopathological results were not yet in but the probability of cancer was high. This scene was built around delivering bad news. In the second scene, the patient insisted on getting the results before a business trip abroad and believed that the practitioner was reluctant to give it to her (this scene was about confidence in the physician), and in the third scene, the patient was increasingly insistent and aggressive about getting the results (this scene consisted in dealing with the patient’s emotion and aggressivity). In the second scenario, the first scene consisted of a practitioner’s announcement of colon cancer with liver metastasis and the patient’s denial of the diagnosis (this scene was about the understanding of denial as a protective psychological mechanism). In the second scene, the patient accepted the diagnosis but was reluctant to accept the treatment, which required the practitioner’s appropriate and detailed explanations (this scene was about dealing with the fear of treatment and adverse effects), and in the third scene, the patient emotionally collapsed at the announcement of treatment, crying because she was reminded of her mother, who had died of this cancer despite treatment (this scene consisted in dealing with emotional collapse). In the third scenario, the first scene consisted of the practitioner’s explanation of the transition from curative to palliative care and the patient’s request for a second opinion as she still hoped for a cure (this scene was about dealing with prognosis acceptance and hope). In the second scene, the patient expressed anger toward the physician (this scene was about the understanding of anger as a protective psychological mechanism), and in the third scene, the patient requested euthanasia from the physician (this scene was about the moral dilemma around euthanasia).

The other FT session consisted of three different scenarios. The first scenario was a practitioner’s home visit to an elderly person who had fallen and been discovered by her housekeeper. As the likely diagnosis was a femoral neck fracture, hospitalization was recommended. In the first scene, the patient refused because of poor explanations given by a rude physician in a hurry (this scene was about wrong communication); in the second scene, the patient refused for apparently no reason, and appropriate questioning showed that it was related to her husband, who had died in the hospital (this scene was about the understanding of resistance as a protective psychological mechanism); and in the third scene, the housekeeper was very stressed and therefore constantly interrupted the exchanges between the elderly women and the physician, making disturbing remarks (this scene consisted in dealing with a disruption in communication with the patient). The second scenario was a medical appointment with a patient suffering from depression. In the first scene, the patient had obvious signs of depression but no suicidal ideation, and her state was minimized by a rude doctor (this scene was about wrong communication). In the second scene, she evoked suicidal ideas and requested prompt care. In the third scene, she expressed strong suicidal ideation and requested immediate hospitalization (these scenes consisted in the assessment of a suicidal risk in a near-mutic patient). The third scenario was the announcement of type 1 diabetes to an adolescent and his mother. In the first scene, the doctor gave unclear pathophysiological explanations about diabetes, without checking whether the adolescent and his mother were following these explanations (this scene was about wrong communication); in the second scene, the concerned mother constantly interrupted the doctor’s exchange with the adolescent with questions regarding the immediate and long-term risks of diabetes (this scene consisted in dealing with a disruption in communication with the patient); and in the third scene, the mother emotionally collapsed at the announcement, crying in front of her son (this scene consisted in dealing with emotional collapse).
